# Development and Validation of UPLC-MS/MS Method for Determination of Enasidenib in Rat Plasma and Its Pharmacokinetic Application

**DOI:** 10.1155/2020/5084127

**Published:** 2020-03-31

**Authors:** Shuang-long Li, Yong-liang Zhu, Yi Zhang, Shu-han Liu, Xiang-die Wang, Xiang-jun Qiu

**Affiliations:** Medical College of Henan University of Science and Technology, Luoyang 471023, China

## Abstract

In our research, a straightforward UPLC-MS/MS method, with diazepam as the internal standard (IS), was proposed and acknowledged to determine the concentrations of enasidenib in rat plasma. When preparing the sample, we used acetonitrile for protein precipitation. The gradient elution method was used, and the mobile phase was acetonitrile and 0.1% formic acid. Diazepam was used as the IS. We used the Acquity UPLC BEH C18 column to separate enasidenib and IS. Under the positive ion electrospray ionization (ESI) source conditions, the mass transfer pairs of enasidenib were monitored by multiple reaction monitoring (MRM) to be *m/z* 474.2 ⟶ 456.1 and *m/z* 474.2 ⟶ 267.0, and the IS mass transfer pairs were *m/z* 285.0 ⟶ 154.0. Enasidenib had good linearity (*r*^2^ = 0.9985) in the concentration range of 1.0–1000 ng/mL. Besides, the values of intraday and interday precision were 2.25–8.40% and 3.94–5.46%, respectively, and the range of the accuracy values varied from −1.44 to 2.34%. Matrix effect, extraction recovery, and stability were compliant with FDA approval guidelines in terms of bioanalytical method validation. We had established a new method that had been applied to the pharmacokinetic study of enasidenib in rats.

## 1. Introduction

Acute myeloid leukaemia (AML) is a disease of bone marrow hematopoietic stem cells [1]. AML was typically characterized by the accumulation of immature myeloid cells in the bone marrow and inhibition of bone marrow hematopoiesis [2]. The clinical manifestations of AML were anemia, hemorrhage, infection, fever, organ of infiltration, and so on. The condition was acute and the prognosis was dangerous, and it was often life-threatening [3]. The current main treatment strategies for AML include intensive chemotherapy, nonintensive chemotherapy, and small molecule inhibitors [4]. However, the overall effect of these treatments was not good, and it was necessary to further develop new treatment methods of the disease.

**Figure 1:**
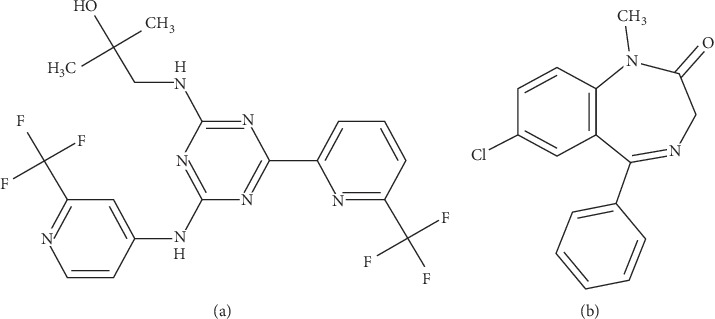
The chemical structure of enasidenib (a) and diazepam (b) in the present research.

Enasidenib (Figure 1(a)) was a small molecule isocitrate dehydrogenase-2 (IDH2) inhibitor and was approved in the USA on 1 August 2017 for the treatment of patients with relapsed or refractory AML and IDH2 mutations [5, 6]. The half-life of enasidenib after oral administration of 100 mg was approximately 137 hours [7]. Its plasma exposure (AUC) increased proportionally with the daily dose of 50–450 mg. In addition, age, weight, race, mild liver damage, and kidney damage were thought to alter the pharmacokinetics of enasidenib [8–10].

Now, enasidenib has provided a new treatment approach for patients with refractory AML with recurrence and IDH2 mutations [11–13]. However, there are only three papers which reported the pharmacokinetic profiles of enasidenib [14–17]. Among them, only one LC-MS/MS method has been published to determine the concentration of enasidenib in biological media in detail, which has long analytical time, low sensitivity, and complex sample preparation process [16]. It is believed that detecting enasidenib in plasma would help to establish the properties of pharmacokinetics and investigate the drug-drug interactions in animal models. In this article, a quick, straightforward, and accurate UPLC-MS/MS method was proposed and acknowledged to quantify enasidenib level and had been applied to the pharmacokinetic study of enasidenib.

## 2. Materials and Methods

### 2.1. Chemicals

Enasidenib (>98%) was obtained from the Beijing Sunflower Technology Development CO., Ltd. Diazepam (>98%, IS, Figure 1(b)) was bought from Sigma (St. Louis, MO, USA). Formic acid was purchased from Anaqua Chemicals (Wilmington, USA). Acetonitrile and methanol were both of chromatographic grades.

### 2.2. Instrumentation

The analysis used a Waters UPLC system (Milford, MA, USA), and the separation column used an Acquity BEH C18 column (2.1 mm × 100 mm, 1.7 *μ*m) with gradient elution. The mobile phase consisted of 0.1% formic acid (A) and acetonitrile (B) with the flow rate of 0.30 mL/min. The column temperature was set as 45°C. The scheme of gradient elution was implemented as follows: 0–0.5 min (10% A), 0.5–1.0 min (10–90% A), 1.0–2.0 min (90% A), 2.0-2.1 min (90–10% A), and 2.1–3.0 min (10% A). The running time of each sample was set at 3.0 min.

Mass spectrometry was performed on a Waters triple quadrupole mass spectrometer and monitored by MRM in the positive ion ESI mode. The mass transfer pairs were *m/z* 474.2 ⟶ 456.1 and *m/z* 474.2 ⟶ 267.0 for enasidenib and *m/z* 285.0 ⟶ 154.0 for IS. MassLynx 4.1 software (Waters Corp, Milford, MA, USA) was used to control the machine and obtain data.

### 2.3. Preparation of Solutions, Calibration Standards, and Validation Quality Control (QC) Samples

10 mg of enasidenib and IS were accurately measured, respectively, and methanol was added to a 10 ml volumetric flask to prepare 1.0 mg/mL stock solution. The original liquid was gradient diluted by methanol to get massive standard working liquid. Similarly, IS was diluted with acetonitrile to obtain 5 ng/mL IS working solution. QC samples as well as calibration curves were obtained from solutions that surged 10 *μ*L standard or QC work to 90 *μ*L blank rat plasma in polypropylene tubes. The ultimate concentrations of QC samples were 2.0, 400, and 800 ng/mL. The ultimate concentrations of calibration curves were 1.0, 5.0, 10, 50, 100, 200, 500, and 1000 ng/mL for enasidenib.

### 2.4. Sample Preparation

The sample was prepared by protein precipitation. Accurately, 100 *μ*L plasma was drawn to 1.5 mL of the EP tube, and 200 *μ*L of 5 ng/mL IS working solution was added. The sample was vortexed for 1 min and centrifuged at 15,000 ×*g* for 15 min. The supernatant liquid (2 *μ*L) was used for analysis and detection.

### 2.5. Method Verification

The UPLC-MS/MS method was validated in accordance with the guidelines of the United States Food and Drug Administration (FDA, Guidance for Industry: Bioanalytical Method Validation, Rockville, 2018).

The selectivity of this method was evaluated by comparing the chromatograms of plasma from two separate blank rat plasmas, blank plasma in enasidenib and IS, and a plasma sample 1.5 h after oral administration of enasidenib to detect analytes and IS in retention time and endogenous interference.

The linear relationship of this method was determined by a series of concentrations of enasidenib QC samples prepared in triplicate for three consecutive days. The calibration curves were plotted with the peak area ratio of analyte to IS (*y*) against the theoretical concentration (*x*) of each analyte, which contains the weight factor of the reciprocal of the concentration (1/*x*^2^). LLOQ was regarded as the minimum value of the calibration curve when the value of *S*/*N* is more than 10. The carryover test was performed by injecting a blank plasma sample spiked with IS (50 ng/mL) or enasidenib (1000 ng/mL) followed by injecting a blank sample. In this blank sample, each analyte should be less than 20% of the LLOQ.

The precision and accuracy of enasidenib at concentrations of 2, 400, and 800 ng/mL were investigated by repeating 6 times at each concentration for three consecutive days, respectively. The precision was expressed by relative standard deviation (RSD, %) and was calculated from the measured concentration and its true value. The accuracy was expressed as relative error (RE, %), and the RE was calculated by subtracting the true concentration and the true value from the measured value. Both RSD and RE need to be less than 15%.

The extraction recovery and ME of plasma samples were investigated at three concentrations of 2, 400, and 800 ng/mL, respectively, with each concentration repeated six times. The recovery was compared with the peak area of the conventionally pretreated QC sample and the peak area after extraction of the corresponding blank plasma (after extraction). The ME was evaluated by the peak area ratio of the analyte in the sample after extraction and the corresponding water exchange sample.

The stability of QC samples (enasidenib at concentrations of 2, 400, and 800 ng/mL) under 4°C for 12 h, 12 h at room temperature, −20°C for 4 weeks, and −20°C∼25°C for three freeze-thaw cycles was investigated. Both RE and RSD need to be less than 15%.

The stock solution stability of enasidenib (1000 ng/mL) at room temperature stability and freeze stability was investigated by six replicates tests. The room temperature stability was achieved by comparing the stock solution stored at room temperature for 24 hours with the remainder of the stock solution stored in a −20°C refrigerator. The freezing stability was achieved by comparing the newly configured stock solution with the stock solution stored in a −20°C refrigerator for 3 months. The solution was considered to be stable if the test value was within acceptable accuracy (RE% ≤±10%) and precision (RSD% ≤15%).

### 2.6. Pharmacokinetic Study

Sprague Dawley rats, with the weight of 200 ± 20 g, were purchased from the Laboratory Animal Centre of Henan University of Science and Technology (Luoyang, China) and the Animal certificate was 2007（Hubei）-0001. The experiment obtained the necessary approval from the Animal Ethics Committee of Henan University of Science and Technology. The experiment was approved according to the Laboratory animals-guidelines for ethical review of welfare (GB/T 35892-2018). The institutional approval number for the preclinical study of this experiment was 2019040013. It was allowed that all animals could freely get water during the research. Eight rats were orally administered 10 mg/kg of enasidenib, and 300 *μ*L of blood was collected from the tail vein of each rat at 0.33, 0.67, 1, 1.5, 2, 3, 4, 6, 9, 12, 12, 24, and 48 hours. The blood samples were centrifuged at 10,000 rpm for 10 min, and the supernatant was frozen at −20°C until analysis. Rat plasma drug concentration data were processed using DAS software (version 2.0).

## 3. Results and Discussion

### 3.1. Method Development

We had established an UPLC-MS/MS method for the determination of enasidenib in rat plasma. This method has high sensitivity and short analysis time (3 minutes).

In order to obtain short retention time and symmetric peak shape, different columns were tested and optimized. The Acquity UPLC CSH C18 column, BEH C18 column, and HSS C18 column (Waters Corp.) were evaluated. The results shown that enasidenib and IS had good separation on the UPLC BEH C18 column. In addition, in order to get the optimal mobile phase, we examined the separation results of acetonitrile, methanol, formic acid, and acetic acid under different conditions. It was observed that acetonitrile and 0.1% formic acid as the mobile phase, better peak shape and higher sensitivity, lower background noise, and shorter retention times of enasidenib and IS could be obtained.

Through comparative screening experiments, diazepam was finally determined as the IS of the experiment. Under the experimental conditions, diazepam was relatively stable, and the retention time was reasonable and does not affect the detection of the test object.

**Figure 2:**
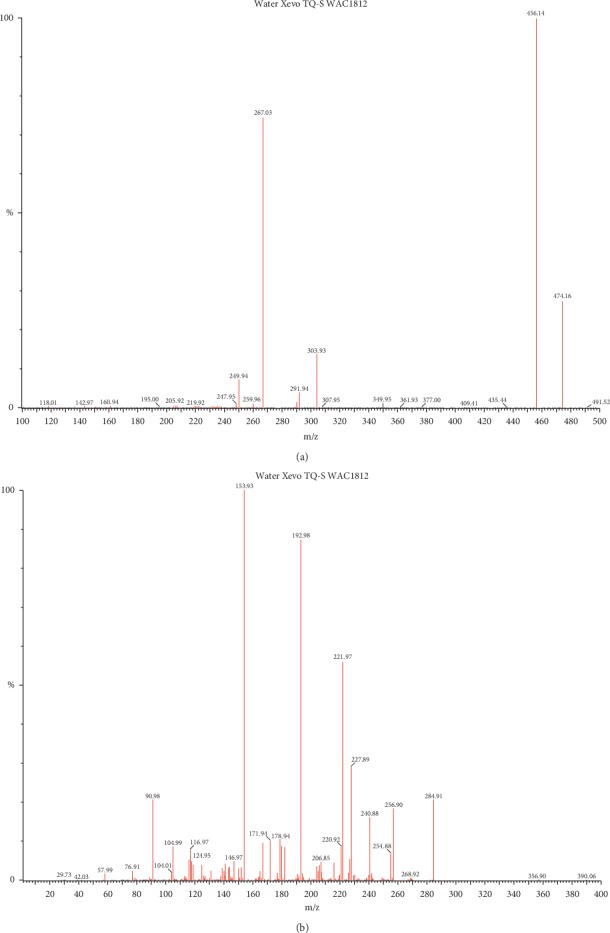
The ion transitions from the parent ion to the daughter ion of enasidenib (a) and diazepam (b).

Multiple reaction monitoring (MRM) was a commonly used analytical method for mass detection. It has the outstanding advantages of strong specificity, high sensitivity, high accuracy, good reproducibility, wide linear dynamic range, and high-throughput automation. In order to optimize the most sensitive ionization mode, we tested positive and negative ions separately. The ion transitions from the parent ion to the daughter ion of enasidenib Figure 2(a) and diazepam Figure 2(b) are shown in Figure 2.

### 3.2. Selectivity

**Figure 3:**
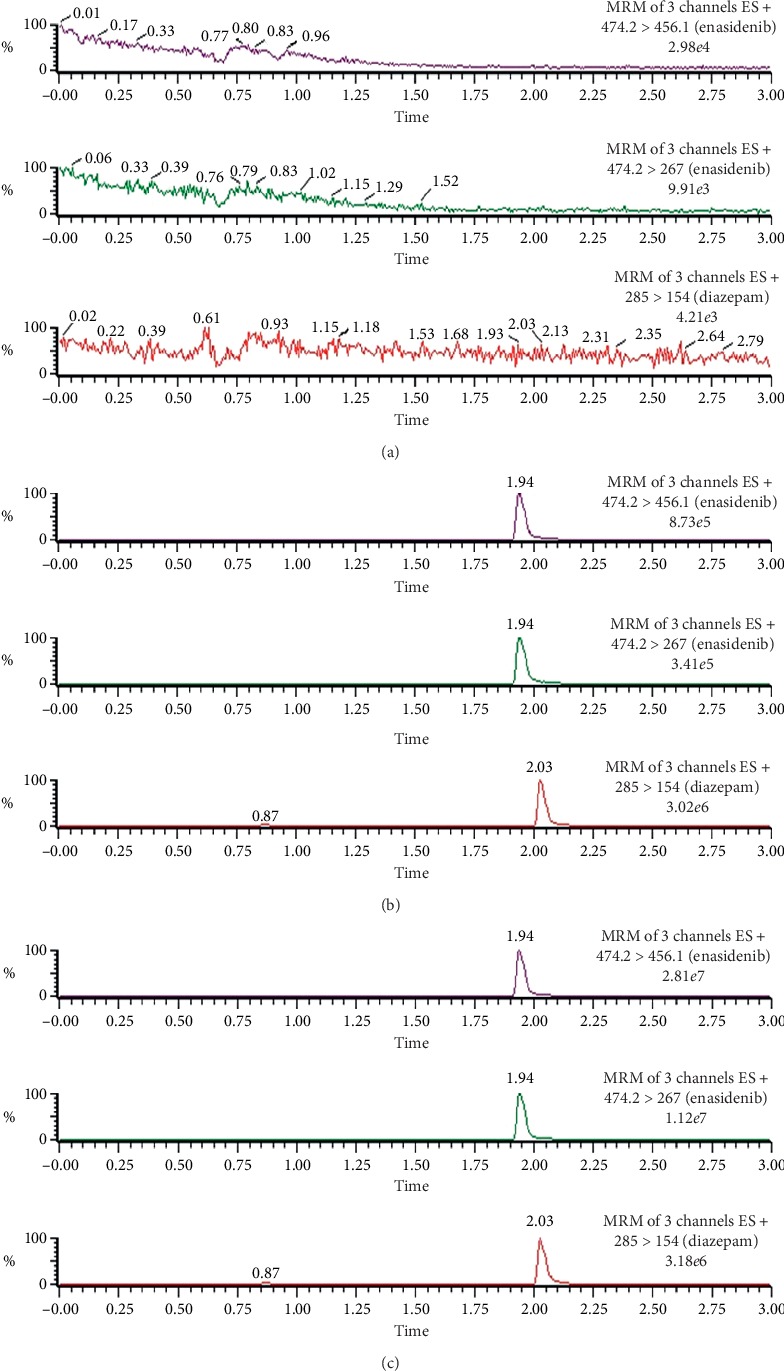
Representative chromatograms of enasidenib and IS in rat plasma samples: (a) a blank plasma sample; (b) a blank plasma sample spiked with enasidenib and IS; (c) a rat plasma sample 1.5 h after oral administration of 10 mg/kg enasidenib.

The specificity of the samples in the three cases was examined. The chromatographic results are shown in Figure 3. It can be seen from the results that the retention times of enasidenib and IS were 1.94 and 2.03 min, respectively. It showed that the UPLC-MS/MS method established in this experiment had high specificity.

### 3.3. Linearity, Sensitivity, and Carryover Effect

The typical calibration standard curve equation of enasidenib in this study is shown as follows: *y* = 10355.8*x* ± 36121.3 and *r*^2^ = 0.9985. In the calibration curve, *y* stands for the ratio of the analyte to the IS peak area, while *x* stands for the analyte's plasma concentration, and the LLOQ was set to 1.0 ng/mL. The results of the carryover test showed that the analyzer did not detect the residual analyte or IS injected into the sample at the next injection. In the UPLC-MS/MS analysis, carryover did not affect the determination of enasidenib and IS.

### 3.4. Accuracy and Precision

The precision (% RSD) and accuracy (% RE) results are shown in Table 1. It can be seen from the experimental results that enasidenib had good precision and accuracy.

### 3.5. Recovery and ME

The recovery and ME results are shown in Table 2. The results showed that, under the chromatographic and MS conditions selected in this study, IS had a good recovery rate, and ME does not affect the determination of enasidenib.

### 3.6. Stability

The stability test results are shown in Table 3. It can be seen that the RE value of enasidenib under the four conditions ranged from −3.45% to 3.75%, with an error of 10%. It can be seen from the experimental results that enasidenib was stable under the experimental conditions.

### 3.7. Stock Solution Stability

Under the experimental conditions, the stock solution stability is shown in Table 4. It can be seen from the experimental results that enasidenib stock solutions were stable.

### 3.8. Pharmacokinetic Study

**Figure 4:**
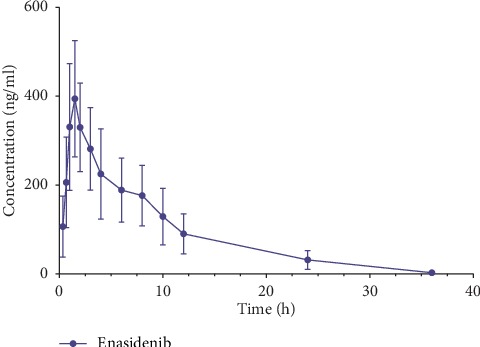
Plasma concentration versus time after oral administration of 10 mg/kg enasidenib in eight rats (mean ± SD).

In a pharmacokinetic study after oral administration in rats, we measured the plasma concentration of enasidenib.  Figure 4 shows the plasma concentrations of enasidenib for time curve in rats after administration, and the main pharmacokinetic parameters are shown in Table 5. *T*_max_ of enasidenib was about 1.57 h, and *t*_1/2_ was about 7.99 h, with a faster peaking time.

## 4. Conclusions

In our study, the method for the determination of enasidenib in rat plasma was established. The method was simple, rapid, specific, and had high recovery. The methods of method validation were in line with the requirements of the bioassay in the guiding principles of pharmacokinetics, providing a convenient, reliable, and stable method for the pharmacokinetic study of enasidenib. This method has been successfully applied to the pharmacokinetics of enasidenib in rats to the pharmacokinetic study of enasidenib in rats.

## Figures and Tables

**Table 1:** Precision and accuracy of enasidenib in rat plasma (*n* = 6).

Analyte	Concentration added (ng/mL)	Intraday		Interday	
		RSD (%)	RE (%)	RSD (%)	RE (%)
Enasidenib	2.5	8.40	−1.20	5.46	0.86
	200	6.48	−1.44	5.38	0.58
	800	2.25	2.34	3.94	−0.30

**Table 2:** Recovery and matrix effect of enasidenib in rat plasma (*n* = 6).

Analyte	Concentration added (ng/mL)	Recovery (%)		Matrix effect (%)	
		Mean ± SD	RSD (%)	Mean ± SD	RSD (%)
Enasidenib	2.5	80.07 ± 2.44	11.4	99.09 ± 13.88	14.01
	400	82.74 ± 2.36	3.1	99.35 ± 5.75	5.79
	800	84.83 ± 1.44	1.8	100.04 ± 1.98	1.98

**Table 3:** The stability of enasidenib in rat plasma (*n* = 6).

Compounds	Spiked (ng/mL)	Room temperature, 12 h		Autosampler 4°C, 12 h		Three freeze-thaw		−20°C, 4 weeks	
		RSD (%)	RE (%)	RSD (%)	RE (%)	RSD (%)	RE (%)	RSD (%)	RE (%)
Enasidenib	2.5	4.51	−0.76	5.94	−3.45	6.79	0.47	8.69	−1.94
	400	5.15	−0.55	3.35	1.96	5.48	0.76	4.08	1.07
	800	2.24	2.73	2.48	3.75	1.35	2.38	2.94	1.79

**Table 4:** The stock solution stability of enasidenib in rat plasma (*n* = 6).

Compounds	Spiked (ng/mL)	Room temperature, 12 h		−20°C, 3 months	
		RSD (%)	RE (%)	RSD (%)	RE (%)
Enasidenib	1000	2.88	0.59	3.66	0.94

**Table 5:** Pharmacokinetic parameters of enasidenib in rats after 10 mg/kg oral administration (*n* = 8).

Parameters	Enasidenib
*t*_1/2_ (h)	7.99 ± 3.29
*T*_max_ (h)	1.57 ± 0.73
*C*_max_ (ng/mL)	456.80 ± 124.29
AUC_0⟶*t*_ (ng/mL·h)	4754.06 ± 1685.42
AUC_0⟶∞_ (ng/mL·h)	4929.53 ± 1947.91
*V*_*z*_/*F* (L/kg)	24.91 ± 9.80
CL_*z*_/*F* (L/h)	2.34 ± 1.01
MRT_0⟶*t*_ (h)	12.85 ± 1.50
MRT_0⟶∞_ (h)	12.88 ± 1.06

Abbreviations: *t*_1/2_, half-life; *T*_max_, time of peak concentration; MRT_(0⟶*t*)_, mean residence time of 0 ⟶ *t* time; MRT_(0⟶∞)_, mean residence time of 0-infinity time; *C*_max_, peak concentration; AUC_(0⟶t)_, area under the curve of 0 ⟶ t time; AUC_(0⟶∞)_, area under the curve of 0 ⟶ infinity time; *V*_*z*_/*F*, apparent volume of distribution; and CL_*z*_/*F*, clearance.

## Data Availability

The data used to support the findings of this study are available from the corresponding author upon request.
